# Benzodiazepine (BZD) Use and Patient Safety: Opportunities for Community Pharmacy Involvement in the Management of Drug Interactions

**DOI:** 10.3390/pharmacy13060181

**Published:** 2025-12-11

**Authors:** Juan Ramón Santana Ayala, Daida Alberto Armas, Veronica Hernández García, Armando Aguirre-Jaime, Ángel J. Gutiérrez, Soraya Paz-Montelongo, Arturo Hardisson de la Torre, Carmen Rubio Armendáriz

**Affiliations:** 1 Environmental Toxicology and Food and Drug Safety Research Group, La Laguna University, 38071 La Laguna, Santa Cruz de Tenerife, Canary Islands, Spain; 2 Community Pharmacy (Farmacia Lcdo. Juan Ramón Santana Ayala), Av. Guillermo Santana Rivero 13, 35012 Las Palmas de Gran Canaria, Canary Islands, Spain; 3 Independent Biostatistician, 38001 Santa Cruz de Tenerife, Canary Islands, Spain

**Keywords:** benzodiazepines, opioids, interactions, community pharmacy, pharmaceutical care, knowledge

## Abstract

Introduction: During pharmaceutical care, community pharmacists play a crucial role by carrying out interventions aimed at preventing, detecting, and resolving drug-related problems (DRPs) and negative outcomes associated with medication (NOM), simultaneously enhancing patients’ knowledge about their treatments. The chronic use of Benzodiazepines (BZDs) is known to be associated with risks such as tolerance, dependence, and cognitive impairment. Furthermore, the combined use of BZDs with other medications or alcohol may expose patients to significant drug interactions. Objectives: This study aimed to characterize and describe the clinical profile of patients using BZDs, to evaluate the extent of polypharmacy and potential drug interactions, to investigate their level of knowledge regarding BZD treatment, and ultimately, to propose evidence-based interventions from the community pharmacy to contribute to improving patient safety and minimizing risks associated with BZD use. Method: A cross-sectional, descriptive study was conducted in a single community pharmacy in Gran Canaria (Canary Islands, Spain). The study population comprised 125 adult patients with active BZD prescriptions. Data collection was performed through pharmacist–patient structured interviews using a questionnaire that included sociodemographic, clinical, and BZD knowledge variables. Results: Lormetazepam and alprazolam were the BZDs most frequently prescribed and dispensed. Potential drug interactions with other medications were detected in 38.4% of BZD users. Notably, 61.5% of patients using BZDs also reported the concurrent use of opioid analgesics, with tramadol being the most common opioid (48.1% of BZD users were also treated with tramadol). Statistically significant differences were observed between patients with and without BZD and other drug interactions in several adverse outcome variables, including the risk of falls (*p* = 0.003), cognitive impairment (*p* = 0.047), and urinary incontinence (*p* = 0.016). Existing BZD dependence is detected in 25% and 22.1% of cases, respectively. Patients’ knowledge of their BZD treatment revealed critical gaps, which are identified as a challenge and a clear opportunity for intervention through pharmaceutical care services. Conclusions: The findings underscore the essential and proactive role of community pharmacists in identifying and managing drug interactions, as well as in supporting deprescribing strategies through collaborative and interprofessional care models.

## 1. Introduction

Benzodiazepines (BZDs) are widely used in the treatment of mental and emotional health disorders [1,2]. Since their introduction in the 1960s, they have been valued for their efficacy and safety [3]. However, their chronic use, especially in older adults, has been associated with significant risks, such as tolerance, dependence, cognitive impairment, and increased risk of falls [4,5,6,7]. 

Globally, approximately 10% of the adult population in developed countries has used BZDs, with a higher prevalence in women and older adults [8]. In Europe, Spain ranks among the countries with the highest prescription rates for BZDs in the European Union [9,10]. In addition, Spain leads the world in the consumption of these drugs, with 9.7% of the adult population reporting their use in the last 30 days [11,12]. During the COVID-19 pandemic, the consumption of hypnotics in Spain, including BZDs, increased significantly, reaching 93 defined daily doses per 1000 inhabitants, 6% more than in 2019 [13]. This high consumption reflects both social problems, mainly loneliness and anxiety, and deficiencies in the healthcare system itself, where medicalisation has become the most accessible response to addressing these emotional problems [14].

It has been reported that the combination of BZDs with opioids, sedative antidepressants, neuroleptics, anticonvulsants, antihistamines, or alcohol intensifies the sedative and central nervous system depressant effect of BZDs, increasing the likelihood of serious adverse events [15,16]. Due to these risks of drug interactions, it is essential that their use be carefully monitored in clinical practice, especially in polymedicated patients or those undergoing prolonged treatment [17,18].

Although BZDs and opioids have different therapeutic applications, their combined use has become widespread and is a growing concern. As they have a similar action, their combination potentiates the depressant effects of BZDs, increasing the risk of negative outcomes associated with medication (NOM), like excessive sedation, cognitive impairment, and a higher risk of falls, especially in older people, as well as a greater likelihood of dependence and abuse. In addition, the BZD-opioid combination can have negative effects on the respiratory and cardiovascular systems, posing a significant risk of overdose and mortality [19,20,21].

The intervention of community pharmacists in detecting drug-related problems (DRPs) such as interactions with BZDs can contribute to improving the safety of BZD use [22]. A DRP has been defined by the Pharmaceutical Care Network Europe (PCNE) [23] as “an event or circumstance involving drug treatment that actually or potentially interferes with the patient experiencing an optimum outcome of medical care”. A systematic review by Gray et al. (2023) [24] evaluating pharmaceutical interventions aimed at improving medication use found that pharmaceutical care achieves a 35% reduction in the risk of adverse reactions. Szilvay et al. (2021) [25] estimated that community pharmacies in Hungary resolve 88.6% of drug interactions, demonstrating the value of community pharmacists in ensuring treatment safety and minimizing risks to patients.

The role of community pharmacists has evolved beyond the mere dispensing of medicines, becoming key agents in comprehensive healthcare [26]. In many countries, their role is increasingly patient-oriented, offering Clinical Professional Pharmacy Services that improve, among other things, safety, efficacy, and adherence to treatments [27,28]. In addition, collaborative practice between pharmacists and other healthcare professionals has been strengthened through applied research initiatives, enabling the development of more effective strategies to identify and prevent risks associated with medication use [29]. 

For all the above reasons, including the recognized public health concern regarding benzodiazepine (BZD)’s inappropriate use and the critical role of the community pharmacist, this study aims to investigate BZD use within a community pharmacy setting. Specifically, the objectives of this research are to:

- Characterize and describe the clinical and sociodemographic profile of patients using BZDs.
- Evaluate their polypharmacy status and potential drug–drug interactions.
- Detect patients with existing contraindications for BZD use.
- Describe the adverse effects of BZDs as perceived and reported by patients.
- Explore the patients’ current knowledge base regarding their BZD treatment.
- Propose and delineate targeted interventions from community pharmacists that contribute to improving safety and minimizing risks associated with BZD use.

## 2. Method

Cross-sectional descriptive study with an analytical component, single-center and without a control group, based on a pharmaceutical care protocol during the dispensing of benzodiazepines in a community pharmacy (Gran Canaria, Canary Islands, Spain).

### 2.1. Sample

The sample size (125 patients) was estimated to ensure adequate statistical representativeness, considering retrospective dispensing data for this group of drugs at the study center (community pharmacy in Gran Canaria, Canary Islands, Spain). The study included all patients over 18 years of age, of both sexes, with an active medical prescription for BZD, who came to the community pharmacy requesting the dispensing of a single-drug BZD medication and who had no impairments in communication or decision-making abilities. All participants received detailed information regarding the study protocol and provided informed consent forms. Patients who did not agree to participate in the study, did not meet age or communication ability criteria, had prescriptions for non-single-drug BZDs containing more than one BZD in their composition, or were pregnant or breastfeeding were excluded.

### 2.2. Procedure, Data Collection and Variables Under Study

After identifying patients eligible for the study in the dispensing service, patients were referred to the pharmacist responsible for the study for an interview following a specific data collection questionnaire with 77 questions and several validated tests, such as the Morisky–Green test to assess adherence to BZD treatment, the Tyrer test to assess the risk of BZD dependence, and the EQ-5D-5L questionnaire.

In cases where the patient was unable to respond on their own, support from their caregiver was allowed. The data collected was recorded in an anonymized and confidential database. During the interview and as part of the pharmaceutical care, not only was health education offered to strengthen the patient’s knowledge about the BZD they were using, but the possibility of deprescribing BZD treatment was also explained. Drug interactions were identified using the pharmacy management software linked to BOT PLUS, the official Spanish medicines database (CGCOF), and validated through the pharmacist’s professional judgement [30].

The variables under study were grouped into blocks: sociodemographic, pharmacotherapeutic/clinical, and knowledge about BZDs.

### 2.3. Statistical Analysis

Data analysis was performed using SPSS 30.0. 0TM software from IBM Co.^®^ (Armonk, NY, USA) on a Windows NT 365 Professional operating system from Microsoft Co.^®^ (Redmond, WA, USA), considering a statistical significance level of *p* ≤ 0.05 in all hypothesis tests. Qualitative variables were analyzed using the Chi-square test, or Fisher’s exact test when the number of cells with an expected count of less than five represented more than 20% of the total. For numerical variables without normal distribution, the Mann–Whitney test was applied. In addition to descriptive analysis, patients with and without interactions were compared, and subgroups of interest, such as BZD–opioids, were explored.

## 3. Results

### 3.1. Sociodemographic Profile and Substance Use History

Table 1 presents the sociodemographic profile of the 125 patients included in the study, grouped into two: patients with (38.4%) and without (61.6%) detected interactions between BZD and other treatments. When comparing the group of patients with positive BZD–other drug interactions with those without drug interactions, the distribution by sex is similar, with a predominance of females in both groups.

Analysis of the history of substance use disorders in our sample reveals that the group of patients with positive BZD–other drug interactions had a higher prevalence of previous alcohol consumption (6.3%) and other psychoactive substance use (16.7%) than the group without BZD–other drug interactions (alcohol consumption: 3.9%; other substance use: 9.1%) (Table 1).

### 3.2. Polypharmacy and Drug–Drug Interactions

Of the total sample of 125 patients, 38.4% had some interaction between BZDs and other drugs. The polymedication of our patients has allowed us to detect several patients with more than one simultaneous interaction (5 patients present 2 interactions, and 1 patient suffers 3 interactions) out of a total of 52 recorded interactions.

**Figure 1:**
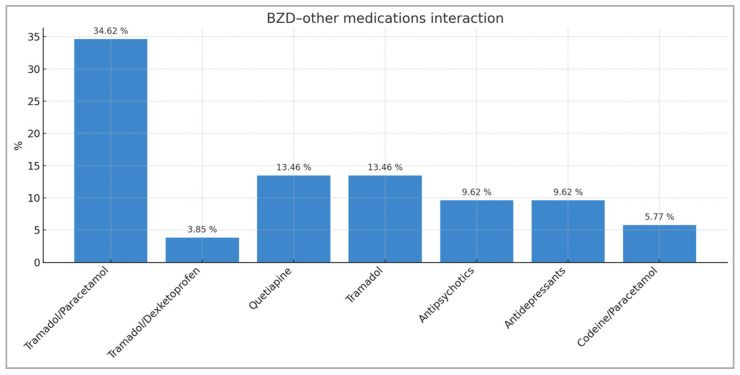
Distribution of BZD–other active drugs/treatment interactions with percentages representing the total number of interactions recorded (n = 52).

Opioid medications account for 61.55% of all interactions detected in patients using BZDs. Tramadol, alone or in combination, accounts for 48.09% of all BZD–other medication interactions and 66.67% of all BZD–opioid interactions. The major opioid group (tapentadol, fentanyl, buprenorphine/naloxone) accounts for 13.46% of interactions detected in patients treated with BZDs. Other relevant interactions observed are those between BZD and quetiapine (13.46%), antipsychotics (9.62%), antidepressants (9.62%), and codeine/paracetamol (5.77%) (Figure 1).

**Figure 2:**
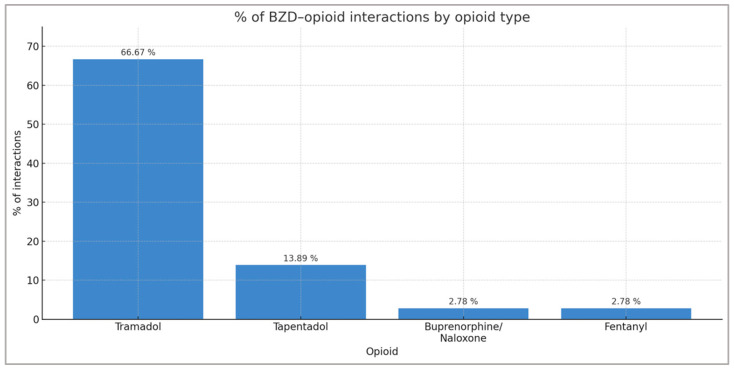
Distribution of BZD–opioid interactions by opioid type.

When the same comparison is made with BZD–opioid interactions by type, it can be seen that the opioid that causes the most interactions with BZD is tramadol (66.67% of all BZD–opioid interactions), followed by tapentadol (13.89%), buprenorphine/naloxone (2.78%), and fentanyl (2.78%) (Figure 2).

### 3.3. Adverse Effects Reported by Patients

Table 2 shows the prevalence results for each of the adverse effects that the studied BZD users report experiencing. Urinary retention, cognitive impairment, urinary incontinence, and falls show statistically significant differences. Specifically, falls were reported by 31.3% of patients with interactions, compared with 10.4% of those without (*p* = 0.003). Urinary retention occurred in 20.8% versus 3.9% (*p* = 0.05), and urinary incontinence in 25.0% versus 9.1% (*p* = 0.016). Likewise, cognitive impairment was more frequent among patients with BZD–other drug interactions (35.4%) than among those without (19.5%) (*p* = 0.047).

Excessive sedation and dysarthria were more frequently reported by patients with BZD–other drug interactions (12.5% and 35.4%, respectively) compared to patients without interactions (7.8% and 7.8%, respectively). A higher percentage of paradoxical disinhibition and erectile dysfunction was also reported by patients with positive BZD–other drug interactions (20.8% and 12.5%, respectively).

The assessment of patient knowledge concerning BZD treatment and its associated risks demonstrates significant findings when comparing the two groups of patients under study (BZD users with positive BZD–other drug interactions vs. BZD users without interactions) (Table 3).

### 3.4. Patient Knowledge, Treatment Effectiveness, and Contraindications

**Figure 3:**
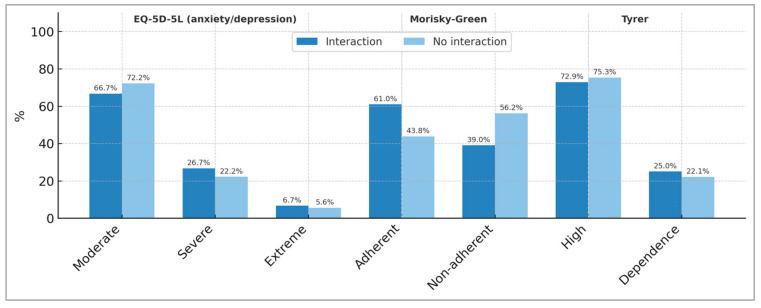
Results of the EQ-5D-5L (anxiety/depression dimension), Morisky–Green, and Tyrer tests in the two groups of Benzodiazepine-using patients studied (patients with detected interactions and without detected interactions due to Benzodiazepines).

The reason for treatment with BZDs that patients report knowing is predominantly anxiety and insomnia and is similar between both groups of patients (Table 3). However, the results of the anxiety/depression dimension of the EQ-5D-5L test show that for both groups of patients (with and without BZD-other drug interactions), a high percentage of BZD users continue to suffer from moderate depression/anxiety (66.7% among patients with BZD and other drug interactions vs. 72.2% among patients without drug interactions). We are concerned about the effectiveness of BZD treatment in patients who report continuing to suffer from high anxiety/depression (26.7% vs. 22.2%) and extreme anxiety/depression (6.7% vs. 5.6%) (Figure 3).

Significant deficiencies in patient knowledge of BZD treatment are observed (Table 3) in relevant aspects such as dosage, duration of treatment, contraindications, adverse effects, interactions of BZD with other treatments, and the risk of developing both tolerance and withdrawal syndrome to BZD. Knowledge of withdrawal syndrome stands out as statistically significant (*p* = 0.009) and higher in the group of patients without BZD–other drug interactions.

With regard to BZD dosage, it is noteworthy that 25% of patients who experience some interaction between BZD and other medications report knowing the BZD dosage to be taken, but the community pharmacist detects that the dosage taken by the patient does not match the prescribed dosage (Table 3).

The results of the Morisky–Green test, which measures therapeutic adherence among our patients who use BZD, are shown in Figure 3. The percentage of patients not adhering to BZD treatment was 39.0% in the group with positive BZD–other drugs interactions and 56.2% in the group without interactions.

**Figure 4:**
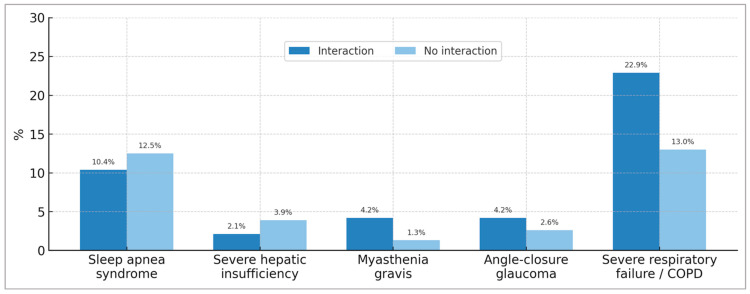
Percentage of patients using benzodiazepines despite presenting relevant clinical contraindications, with and without drug interactions. COPD: Chronic Obstructive Pulmonary Disease.

It is noteworthy that the patients interviewed were largely unaware of the contraindications for BZD use (Table 3). Thus, 52.1% of BZD users with interactions and 54.5% of BZD users without interactions stated that they were unaware of the contraindications. When investigating BZD treatment in patients with contraindications for its use, we observed significant figures. Five contraindications of particular interest for BZD use were explored: sleep apnea syndrome, severe hepatic impairment, myasthenia gravis, narrow-angle glaucoma, and severe respiratory failure/severe COPD (Chronic Obstructive Pulmonary Disease) (Figure 4). Respiratory failure/severe COPD was found to be the most prevalent contraindication, reaching 22.9% among BZD users with BZD–other drug interactions and 13.0% among BZD users without drug interactions. This is followed by the contraindication for sleep apnea, with prevalences of 10.4% and 12.5% for these same patient groups, respectively.

The results on knowledge of the risk of developing BZD withdrawal syndrome are noteworthy (Table 3). The observed statistical significance (*p* = 0.009) reinforces the need for community pharmacies to instruct BZD users on how to observe and detect signs and symptoms of withdrawal.

The risk of BZD dependence was investigated using the Tyrer test [31] (Figure 3). The results show that, for both groups of patients (with detected and undetected BZD–other drug interactions), there is a high risk of BZD dependence (72.9% and 75.3%, respectively), and, in addition, existing BZD dependence is detected in 25% and 22.1% of cases, respectively.

This limited knowledge among BZD users likely correlates with the high percentage of patients who have not read the package insert (47% among BZD users who stand out for presenting BZD–other drug interactions and 50.6% among BZD users without interactions with other drugs) (Table 3).

## 4. Discussion

The risks associated with BZD use, including cognitive impairment, chronic insomnia, daytime sleepiness, and functional limitations, are well-documented in the literature, supporting the need for strict follow-up and short treatment durations [5,32]. This is compounded by the known additive effect of various Central Nervous System (CNS) depressants and the anticholinergic burden generated by certain interactions, which lead to significant risks such as postural instability, falls, cognitive impairment, and urinary tract dysfunction [33,34]. Furthermore, the combined use of BZDs with alcohol and other psychoactive substances is a widely known concern [35,36,37]. Abuse of BZDs occurs almost exclusively among subjects with co-occurring substance abuse issues due to cross-dependence [38,39]. Consistent with the existing literature, which identifies BZD–opioid, BZD–antidepressant, and BZD–antihistamine combinations as the most frequent interactions [40], our findings underscore the high prevalence of concurrent prescribing.

The combined use of opioids and BZDs is a recognized global public health concern due to the synergistic risk of Central Nervous System (CNS) depression. This public health challenge is evident in our results, which highlight the elevated consumption of opioid medications—specifically tramadol—among our BZD-using patients. Globally, studies reflect this increasing risk, particularly among older adults (aged 65 and over) in the United States [20]. Crucially, our study reinforces the clinical relevance of this association: the prevalence of falls was significantly higher in patients with BZD–other drug interactions (31.3%) compared to those without interactions (10.4%). This aligns with the findings of Maust et al. (2022) [41], who observed that the co-prescribing of opioids or antipsychotics with BZDs increases fall injuries within the first 10 days, thus strengthening the demonstrated relationship between the concomitant use of BZDs and other psychotropic drugs and the increased risk of adverse events like falls. Our results, which are methodologically similar to those of Sánchez-Valle et al. (2024) [42], strongly support the need for targeted intervention.

Beyond the risks of interaction, our findings also touch upon treatment effectiveness. In these BZD-using patients where anxiety and depression persist (similar to rates reported by García et al., 2021) [43], the community pharmacy must be prompted by the suspicion of treatment ineffectiveness. This requires an intervention to refer the patient to the prescribing physician for reassessment, thereby addressing the risk of chronic use and potential dependence. Given that abrupt withdrawal can cause rebound symptoms [44], structured withdrawal with monitoring is necessary [3]. 

These results invite us to reorient and enhance the dispensing service of community pharmacies. Our findings reinforce the necessity of designing robust Pharmaceutical Care protocols for BZD users. It is advisable to incorporate these structured protocols directly into the community pharmacy dispensing service for the early detection of underlying or past Psychoactive Substance Use Disorders (SUDs) and for screening at-risk patients, particularly those receiving combined BZD and opioid treatments such as tramadol. This Clinical Professional Pharmacy Service (CPPS) should include a brief assessment of the patient’s knowledge to address any knowledge gaps and prevent potential risks, or even to refer the patient to other services, such as the Medication Review with Follow-up Service. The provision of personalized, accessible information on BZDs by the community pharmacist is essential, whether in verbal or written form or through new technologies [45,46,47]. 

In defense of the value of Pharmaceutical Care, we believe that community pharmacists should intervene proactively in high-risk patients (those with falls, cognitive problems, advanced age, or concomitant drug use) to minimize risks. It is essential to streamline communication with the prescriber to optimize medication use and coordinate patient and treatment monitoring [48,49]. This defense culminates in the promotion of collaborative deprescribing. To avoid the risks of chronic BZD treatment, physicians and pharmacists must work as a team to support the patient in BZD withdrawal [50]. Pharmacist-led BZD deprescribing has been shown to improve outcomes and enhance coordination with the prescriber [51]. Educational interventions led by pharmacists are therefore vital in promoting gradual, monitored discontinuation and preventing abrupt cessation. Taken together, these results reinforce our opinion on the need and appropriateness of a Pharmaceutical Care intervention that evaluates the duration of BZD treatments to verify adherence to clinical guidelines and minimize the risks to patient health arising from treatment dependence.

### Limitations

As this is a single-center observational and descriptive study, it does not describe patterns or allow for the establishment of temporality. The sample size and uneven distribution of some subgroups may have limited statistical power; however, significant associations were observed. The identification of interactions and PIPs (Potentially Inappropriate Prescriptions) was based on the use of structured interviews and review by the pharmacist, which may have generated information bias, potentially leading to under-recording or occasional overestimation. Some of the information was self-reported by the patient, and no follow-up was available to determine the impact of the pharmaceutical intervention. Therefore, the findings should be read as a useful snapshot of actual practice to guide pharmaceutical care in dispensing and coordination with the prescriber. Future studies should include a prospective, multicenter design.

## 5. Conclusions

Few studies have explored patient knowledge and pharmacist-led interventions in this context. The findings underscore the importance of monitoring benzodiazepine treatments, avoiding their combined use with medications that may cause interactions, especially with opioids such as tramadol. In addition, it is essential to promote deprescribing in patients using benzodiazepines and improve their understanding of treatment with these central nervous system depressant drugs. The detection and resolution of negative outcomes associated with benzodiazepines, such as falls, cognitive impairment, and urinary incontinence, should be optimized at all levels of care, given their impact on patients’ quality of life. Therefore, it is necessary to promote collaborative and synergistic communication between healthcare professionals, particularly between prescribing physicians and dispensing pharmacists.

## Figures and Tables

**Table 1:** Sociodemographic profile and psychoactive substance use patterns among benzodiazepine (BZD) users.

	n (%)	Sex	Mean Age (years)	No History of Psychoactive Substance Use Disorder	Patients with Prior Substance Use Disorder	Alcohol Consumption
BZD users with detected BZD–other drug interactions	38.4%	♀: 62.5% ♂: 37.5%	63	77.1%	16.7%	6.3%
BZD users without BZD–other drug interactions	61.6%	♀: 67.5% ♂: 32.5%	63	87.0%	9.1%	3.9%

♀: women; ♂: men.

**Table 2:** Adverse Drug Reactions Reported by Patients to Community Pharmacists During Benzodiazepine (BZD) Treatment.

	BZD Users with Detected BZD–Other Drug Interactions n (%)		BZD Users Without BZD–Other Drug Interactions n (%)		Statistically Significant Differences
	No	Yes	No	Yes	*p* Value
Excessive sedation	42 (87.5%)	6 (12.5%)	71 (92.2%)	6 (7.8%)	---
Erectile dysfunction	42 (87.5%)	6 (12.5%)	71 (92.2%)	6 (7.8%)	---
Ataxia	40 (83.3%)	9 (18.8%)	67 (87.0%)	10 (13.0%)	---
Dysarthria	39 (81.3%)	9 (35.4%)	71 (92.2%)	6 (7.8%)	---
Urinary retention	38 (79.2%)	10 2(0.8%)	74 (96.1%)	3 (3.9%)	*p* = 0.05
Paradoxical disinhibition	38 (79.2%)	10 (20.8%)	69 (89.6%)	8 (10.4%)	---
Urinary incontinence	36 (75.0%)	12 (25.0%)	70 (90.9%)	7 (9.1%)	*p* = 0.016
Falls	33 (68.8%)	15 (31.3%)	69 (89.6%)	8 (10.4%)	*p* = 0.003
Cognitive impairment	31 (64.6%)	17 (35.4%)	62 (80.5%)	15 (19.5%)	*p* = 0.047
Sleep problems	25 (52.1%)	23 (47.9%)	48 (62.3%)	29 (37.7%)	---

**Table 3:** Patient knowledge assessment regarding Benzodiazepine (BZD) therapy.

	Patient Answer Choices	BZD Users with Detected BZD–Other Drug Interactions n (%)	BZD Users Without BZD–Other Drug Interactions n (%)
What diagnosis does the patient believe motivates BZD treatment?	Anxiety	26 (54.2%)	42 (54.2%)
	Insomnia	19 (39.6%)	31 (40.3%)
	Muscle Relaxation	3 (6.3%)	4 (5.2%)
Does the patient know the prescribed BZD dose and does it match what the patient reports taking?	No	23 (47.9%)	39 (50.6%)
	Yes	16 (33.3%)	25 (32.5%)
	Believe they know it, but the reported intake does not match the prescription	12 (25.0%)	13 (16.9%)
Does the patient know the intended duration of BZD treatment?	Do Not Know	1 (2.1%)	7 (9.1%)
	Think It Is Indefinite	22 (45.8%)	46 (59.7%)
	Until The Doctor Says Otherwise	19 (39.6%)	18 (23.4%)
	<3 Months	2 (4.2%)	5 (6.5%)
	>3 Months	4 (8.3%)	1 (1.3%)
Does the patient know the contraindications of the BZD they use?	No	25 (52.1%)	42 (54.5%)
	Know Several	14 (29.2%)	22 (28.6%)
	Know at least 2–4 contraindications	8 (16.7%)	12(15.6%)
	Know > 4 Contraindications	1 (2.1%)	1 (1.3%)
Does the patient know the adverse effects of the BZD they use?	No	25 (52.1%)	41 (53.2%)
	Know Some	15 (31.3%)	19 (24.7%)
	Know Several	7 (14.6%)	16 (20.8%)
	Know Most/All	1 (2.1%)	1 (1.3%)
Does the patient know BZD interactions with other medicines?	No	21 (43.8%)	35 (45.5%)
	Know Some	21 (43.8%)	31 (40.3%)
	Know Several	6 (12.5%)	11 (14.3%)
Does the patient know that BZD treatment can lead to tolerance?	No	22 (45.8%)	30 (39.0%)
	Yes	26 (54.2%)	47 (61.0%)
Does the patient know that BZD treatment can cause withdrawal syndrome, and can they recognize its signs and symptoms?	No	19 (39.6%)	22 (28.6%)
	Yes	29 (60.4%)	55 (71.4%) (*p* = 0.009)
Has the patient read the BZD package leaflet?	No	23 (47.9%)	39 (50.6%)
	Yes	16 (33.3%)	25 (32.5%)
	Partial reading:	9 (18.8%)	15 (19.9%)

## Data Availability

The original contributions presented in this study are included in the article. Further inquiries can be directed to the corresponding authors.
